# Coexistence of a rare type of ectopic kidney with atypical renal vasculature

**DOI:** 10.1007/s12565-020-00584-6

**Published:** 2020-11-03

**Authors:** Anna Waśniewska, Piotr Bukowski, Rafał Szymański, Andrzej Januszewicz, Łukasz Olewnik

**Affiliations:** 1 Department of Normal and Clinical Anatomy, Interfaculty Chair of Anatomy and Histology, Medical University of Lodz, Żeligowskiego 7/9, 90-752 Łódź, Poland; 2 Department of Anatomical Dissection and Donation, Chair of Anatomy and Histology, Medical University of Lodz, Łódź, Poland; 3 Department of Histology, Chair of Anatomy and Histology, Medical University of Lodz, Łódź, Poland; 4 Department of Hypertension, Institute of Cardiology,, Warsaw, Poland

**Keywords:** Crossed fused renal ectopia, Crossed renal ectopia, Renal ectopia, Renal fusion, Renal artery, Renal vein, Vascularization

## Abstract

Knowledge of anatomical anomalies is significant for all specialists in clinical practice and may prevent serious complications following medical procedures. This report presents the rare crossed fused renal ectopia (CFRE) with atypical renal vasculature in cadaver of a 68-year-old man. The ectopic kidney was located on right side with four renal veins, three renal arteries, two ureters, where one of them is double. The embryological background, as well as the potential clinical significance of this morphological variation, is discussed. An interventional radiological and surgical procedure should be appropriately implemented to treat anomalies of vessels and CFRE.

## Introduction

Renal fusion and renal ectopia are congenital abnormalities, and caused disruption of embryological migration of kidney from the pelvic to the renal fossa.(van den Bosch et al. 2010; Rosenthal et al. 2016) Simple renal ectopia occurs when a kidney did not ascend to the renal fossa during embryogenesis and remained in pelvic or other localization. A condition in which a kidney crossed over the midline and is situated contralaterally to its usual localisation is called crossed renal ectopia (CRE). CRE might be with or without kidney fusion. CRE without fusion is an extremely rare abnormality. (van den Bosch et al. 2010; Akdogan et al. 2015) Renal fusion abnormalities occur more frequently in males. (Akdogan et al. 2015; Babu et al. 2015; Shambharkar et al. 2018) Most patients with this anomaly are asymptomatic and diagnosed accidentally during autopsy, radiological or surgical investigation. (Decter 1997; Rosenthal et al. 2016; Shambharkar et al. 2018) Nevertheless, renal fusion or renal ectopia accompany abnormalities of skeletal, genitourinary, cardiovascular and gastrointestinal systems. (Türkvatan et al. 2009; Loganathan and Bal 2019) These anomalies, especially vessel anomalies, may contribute to complications during surgeries and interventional procedures. (Devirgilio et al. 1995; Taghavi et al. 2016; Loganathan and Bal 2019) This report presents a rare case of right-sided crossed fused renal ectopia (CFRE) with atypical vessel formation, four renal veins, three renal arteries, two ureters, where one of them is double.

## Case report

The cadaver of a 68-year-old man was subjected to a routine anatomical dissection for research and teaching purposes at the Department of Normal and Clinical Anatomy of the Medical University of Lodz. The cadaver was a property of the Department of Normal and Clinical Anatomy at the Chair of Anatomy and Histology the Medical University of Lodz, Poland, following a donation to the universities anatomy programme. The dissection was performed in the abdominal cavity. During the dissection, CFRE was noticed. The measurements were taken with an electronic calliper (Mitutoyo Corporation, Kawasaki-shi, Kanagawa, Japan). Each measurement was taken twice by two independent scientists, experienced in anatomical dissection. The measurement accuracy was 0.01 mm. The value and precision of this method had been confirmed in a previous study. (Olewnik et al. 2017).

CFRE was located on the right side between the Th9 and L3 levels of the vertebral column. The length of the kidney was 148.96 mm; the width in the narrowest place, 26.31 mm; and the width in the widest place, 69.31 mm.

### Renal arteries

The right renal artery (RRA) originated from the right side of the Aorta (AO) with the diameter of 12.37 mm. The RRA was divided into four segmental arteries. The diameter of the RRA at division place was 11.62 mm; the diameters of the four segmental arteries at the entrance into the right renal hilum were 4.10, 4.89, 4.40 mm; the last branch of the RRA entered the left renal hilum, and its diameter was 3.39 mm. The left renal artery (LRA) arose from the left side of the AO, in the distance of 26.43 mm below RRA. In that place of origin, the LRA was 5.72 mm in diameter, LRA ran from left to the right side of body under IMA, and caused the narrowing of the LRA. The diameter of the LRA before the IMA was 5.55 mm, under the IMA—4.82 mm, and after the narrowing—7.00 mm. LRA entered the hilum of the ectopic kidney. Below the LRA, Accessory Renal Artery (ARA) arose from the right side of AO. The diameter of ARA in this place was 7.25 mm; the ARA entered parenchyma of the left ectopic kidney.

### Renal veins

**Fig. 1:**
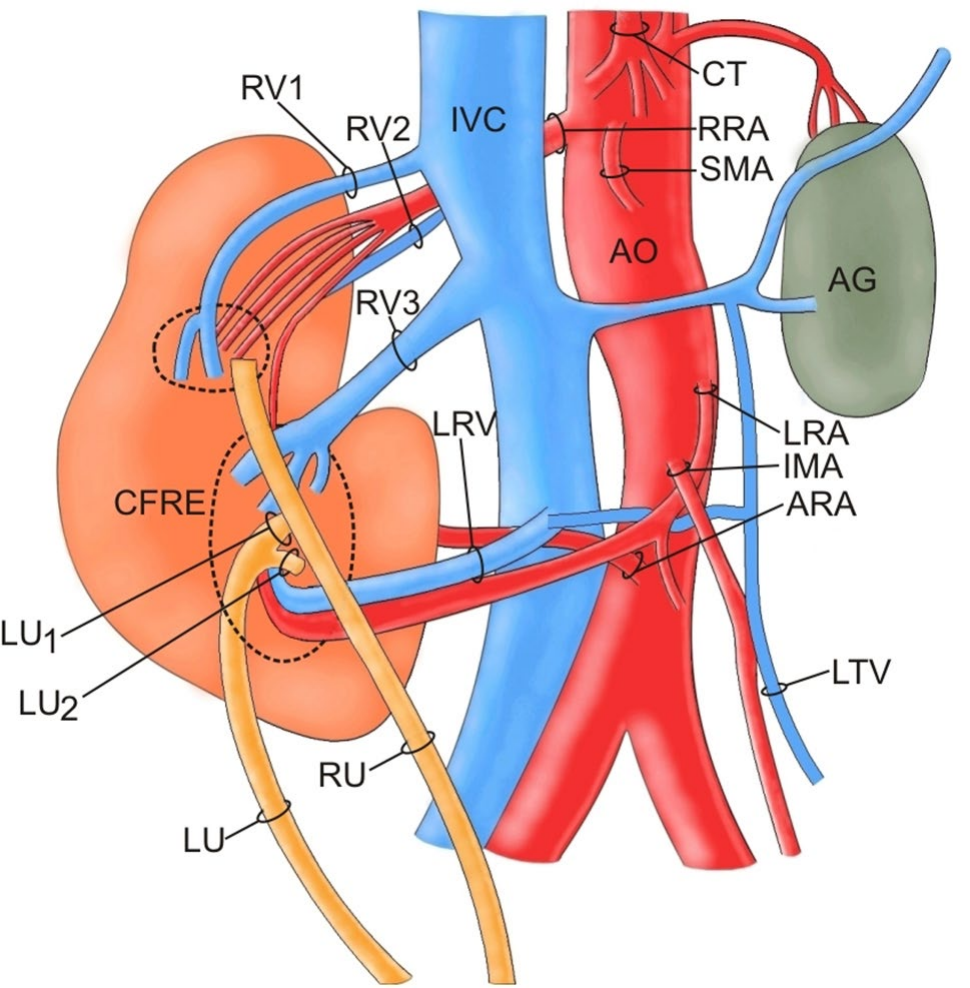
Schema of the abdominal vessels. *AG* Adrenal gland, *Ao* Aorta, *ARA* Accessory renal artery, *CFRE* Crossed fused renal ectopia, *CT* Celiac Trunk, *IMA* Inferior Mesenteric Artery, *IVC* Inferior Vena Cava, *LRA* Left Renal Artery, *LRV* Left renal vein, *LU* Left ureter, *RRA* Right renal artery, *RU* Right ureter, *RV* Renal Vein, *SMA* Superior mesenteric artery

**Fig. 2:**
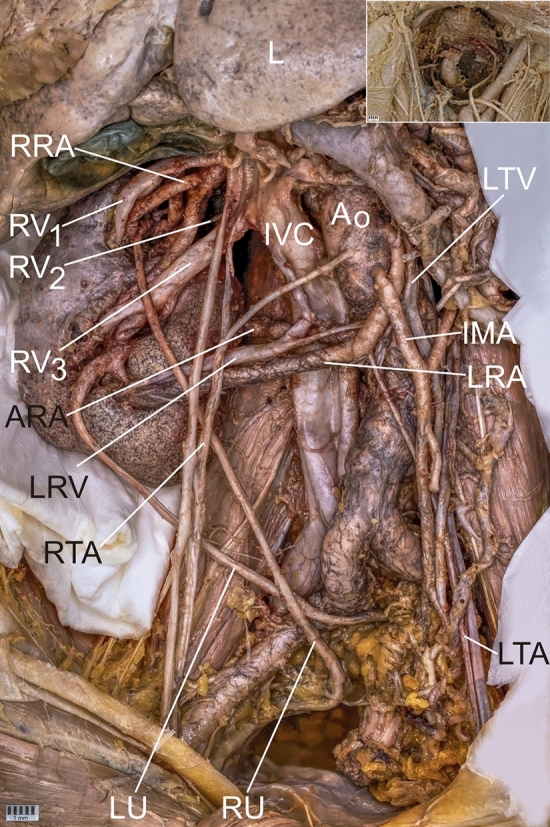
Abdominal vessels and ectopic kidney with the additional perspective of the pelvic cavity in the corner. *AG* Adrenal gland, *Ao* Aorta, *ARA* Accessory renal artery, *CFRE* Crossed fused renal ectopia, *CT* Celiac Trunk, *IMA* Inferior Mesenteric Artery, *IVC* Inferior Vena Cava, *L* Liver, *LRA* Left Renal Artery, *LTA* Left Testicular Artery, *LRV* Left renal vein, *LU* Left ureter, *RRA* Right renal artery, *RTA* Right Testicular Artery, *RU* Right ureter, *RV* Renal Vein, *SMA* Superior mesenteric artery

Blood from the kidney was drained by multiple veins (Figs. 1 and 2 described as RV1–RV3). The vein from the hilum of the left kidney (LRV) run to the Inferior Vena Cava (IVC) and testicular vein (Figs. 1 and 2). The diameter of the LRV at the entrance to the hilum was 3.89 mm, at the division—5.87 mm, at the entrance to the IVC—5.98 mm, and to the testicular vein—2.52 mm.

Three veins which ran from the left hilum created a common trunk (as RV3), and entered the IVC (Figs. 1 and 2). The diameters of these veins after leaving the hilum were, respectively, 3.81, 5.20, 5.68 mm; the diameter of the junction was 9.81 mm; and the diameter at the entrance to the IVC was 12.44 mm.

There was also one separated vein (RV2) which was drained from the right hilum and which entered the IVC. The diameter behind the hilum was 7.02 mm, and at entrance to IVC was 8.93 mm.

Another two veins drained from the upper part of the right kidney hilum, and behind the kidney, and entered the IVC (RV1). The diameters of these veins behind the hilum were, respectively, 5.13 and 5.33 mm; the diameter of the junction was 6.09 mm; and the diameter at the IVC was 11.60 mm.

### Ureters

The ureter of the left ectopic kidney crossed the midline at the level of promontory, and entered the bladder on the left side. The left ureter began as two separated ureters; the length and the width of the shorter part were, respectively, 5.62 mm and 1.57; the length and the width of the longer part were, respectively, 12.37 and 4.45 mm. The length of the ureter behind the junction was 171.53 mm.

Single ureter of the right kidney ran from the right hilum and entered the bladder on the right side. The length and width of the ureter were, respectively, 4.57 and 155.01 mm.

### Adrenal gland

**Fig. 3:**
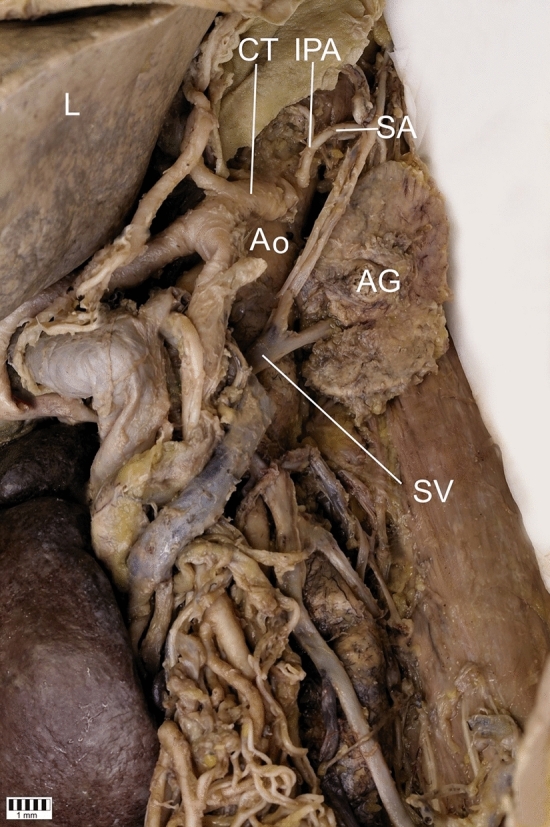
Adrenal gland in the left renal fossa. *AG* Adrenal gland, *Ao* Abdominal Aorta, *CT* Celiac Trunk, *IPA* Inferior Phrenic Artery, *L* Liver, *SA* Suprarenal Artery, *SV* Suprarenal Vein, *RV* Renal Vein

In the left renal fossa, an adrenal gland was located in typical region (Fig. 3). Its structural morphology was histologically confirmed. The length and the width of the adrenal gland were higher than normal with dimensions, respectively, 58.70 and 31.90 mm. Suprarenal artery ran directly from the inferior phrenic artery; suprarenal vein from the adrenal gland drained blood to the IVC. Whereas, right adrenal gland was absent in the typical location and right renal fossa.

## Discussion

Highly diverse congenital anomalies of the kidney and urinary tract (CAKUT) are commonly diagnosed during foetal ultrasound. Nevertheless, CAKUT includes also less frequent, but significant conditions like renal fusion and renal ectopia. (Shambharkar et al. 2018; Lomoro et al. 2019).

CRE occurs in between 0.2 and 0.03% of patients. (Faggioli et al. 2003; Gulas et al. 2018) According to McDonald and McCellan, there are four types of CRE: CRE with fusion (CFRE), CRE without fusion, unilateral CRE (agenesis of one kidney) and bilateral CRE (no fusion between kidneys). (McDonald and McClellan 1957; Akdogan et al. 2015). In CRE, usually, the left kidney moves to the right side. The most frequent is CFRE, which occurs in 85–90% of cases. (Türkvatan et al. 2009; Akdogan et al. 2015) According to literature reports, there are six types of CFRE: inferior CFRE, S-shaped (sigmoid), unilateral lump kidney, disc kidney, L-shaped kidney, and superior CFRE. The ureter of the ectopic kidney in all types of fused kidneys crosses the midline. (Decter 1997; Yin et al. 2014; Liu et al. 2015) The least common is ‘cake’ kidney, which occurs in 2% of renal fusion cases. It is a complete fusion of kidneys in which ureters do not cross the midline. (Türkvatan et al. 2009) Horseshoe kidney (HSK) is a condition where kidneys are fused in the midline. The junction of kidneys is between their lower poles but higher poles remain separated on the opposite sides of the midline. (Taghavi et al. 2016) In our case, the fusion between kidneys is advanced and there is no visible border between renal hila. Hence, it was difficult to classify this case. Nevertheless, the lower lobe is significantly bigger in size than the higher lobe. Besides, one of the ureters crosses the midline, which suggests that it is CFRE, L-shaped kidney.

CFRE is usually concomitant with congenital malformations of other systems, such as: skeletal, genitourinary, cardiovascular and gastrointestinal. (Türkvatan et al. 2009; Rehder et al. 2019; Loganathan and Bal 2019) In this case, it is worth paying attention to the ureter due to its double shape.

In 2014, Yin et al. described renal fusion with “Y”-type fused ureter, and classified it as a new type of CFRE. (Yin et al. 2014) Also Glodny et al. described seven cases with a various number of ureters in fused kidneys. (Rehder et al. 2019) So, is it not worth considering to create another classification of CFRE depending on a malformation of the ureter?

However, multiple anomalies of vascular supply are typical for renal fusion and renal ectopia(Türkvatan et al. 2009; Al-Hamar and Khan 2017). Thus, a structure consisting of three renal arteries and a double ureter in this case is especially unusual. Probably, the key to understanding this situation is nephrogenesis. In 1654, Pamarolus was the first who reported CRE. Nevertheless, embryology of ectopic kidneys is still unclear. (Al-Hamar and Khan 2017; Loganathan and Bal 2019) Normally, kidneys develop between 4 and 5th weeks of gestation, when ureteric bud stimulates metanephric blastema. From 6 to 9th weeks of gestation, kidneys ascend to their final position in the lumbar region. While ascending, kidneys gradually rotate; as a result, renal hila face medially. (Al-Hamar and Khan 2017; Majos et al. 2018; Loganathan and Bal 2019) Compression of umbilical arteries on nephrogenic blastemas might result in renal fusion. Complete fusion of contralateral kidneys, located at the same level, is called ‘cake’ kidney. Horseshoe kidney occurs when only one renal pole is fused. Crossed ectopia, in turn, is a result of permanent compression of umbilical arteries, from the beginning of cranial migration on two metanephric masses. (Al-Hamar and Khan 2017) Genetic and teratogenic factors are also considered to be responsible for these anomalies. (Loganathan and Bal 2019) First, nephrogenic blastemas are supplied by nine pairs of mesonephric arteries which arise from the dorsal aorta. (Gulas et al. 2016) When kidneys ascend to their final position, renal veins and arteries develop into permanent vessels. Development of accessory renal vessels results from changes in blood supply during the renal ascent. (Al-Hamar and Khan 2017).

The crossed ectopic kidney in our case has a multiple supply. One artery enters the kidney through the hilum, one is the branch of the RRA, and the other one enters through the renal capsule. Al- Hamar and Kahn described a similar case of CRE; however, there was no fusion between kidneys. (Al-Hamar and Khan 2017) Also, Majos et al. reported that accessory renal arteries of horseshoe kidney tend to avoid the hilum and directly enter parenchyma. (Majos et al. 2018).

According to Iwanaga et al., ectopic kidney may inhibit the development of gross vessels. They reported L- shaped kidney with multiple vascular anomalies. In their case, RRA arose from the AO from the right side and the LRA from its left side, which is similar to our case. However, in their case, there were three surplus renal arteries, two of which arise from the AO and one from the bifurcation point of common iliac arteries. The RRV, LRV and one surplus renal vein from the right renal hilum were drained into the IVC. However, there was no right common iliac vein, which, in the authors’ opinion, is associated with ectopic kidney. (Iwanaga et al. 2017) Pupca et al. reported L-shaped CFRE with two LRVs with double nutcracker syndrome, where the first vein was compressed between the superior mesenteric artery and the aorta, and the second vein was compressed between the aorta and the L2 vertebral body. (Pupca et al. 2014).

Renal artery entrapment is a compression on the renal artery, which can be manifested by: low renal perfusion, hypertension or nephropathy. Most cases of renal entrapment are results of musculotendinous fibres, hypertrophic diaphragmatic crus or high ectopic renal artery origin. (Arazińska et al. 2016) This case is extraordinary because the left renal artery is compressed by the inferior mesenteric artery. The patient may not have suffered any symptoms due to adequate perfusion of the kidney, being a consequence of fusion and presence of two other arteries.

An adrenal gland abnormality poses another problem in our case. First, the right side was involved by CFRE and we noticed only one adrenal gland, situated on the left side. Second, the width of the adrenal gland was 31.90 mm and its length—58.70 mm which means it was bigger than a healthy adrenal gland, which is on average 30.00 mm wide and 50.00 mm long. (Carlos et al. 2006) Although the adrenal gland is a highly significant key component of the stress system, its morphology is rarely described in literature. (Kanczkowski et al. 2017) Adrenal ectopia or adherent adrenals and their unusual vascular anomalies may result in difficulties during adrenalectomy. (Donnellan 1961) That is why, a new classification of CFRE should also include the changes in the adrenal position and its vascular supply.

Rare symptoms of CFRE are related to calculus formation, hydronephrosis or infection.(Türkvatan et al. 2009; Gulas et al. 2018) Nevertheless, most cases of this anomaly are asymptomatic and diagnosed accidentally during autopsy, radiological or surgical investigation.(Decter 1997; Mudoni et al. 2017; Shambharkar et al. 2018) Hence, CFRE is a great challenge for surgeons. (Devirgilio et al. 1995; Loganathan and Bal 2019) Fusion of kidneys is the most common anomaly noticed during an aortic surgery. (Glodny et al. 2009) Since the anomaly is not frequent, there are no customary procedures for treating associated carcinoma or stones. (Cao et al. 2019) These two arguments call for conducting more extensive studies on CFRE.

## Conclusions

We reported a rare case of right-sided crossed fused renal ectopia with atypical vessel formation, four renal veins, three renal arteries, two ureters, one of which is double. Although CFRE is mainly asymptomatic, it is of high clinical significance due to its arterial and venous abnormal course. An interventional radiological and surgical procedure should be appropriately implemented to treat anomalies of vessels and CFRE.
